# (*Z*)-3-(4-Bromo­anilino)-1-ferrocenylbut-2-en-1-one

**DOI:** 10.1107/S1600536810047628

**Published:** 2010-11-24

**Authors:** C. Valdebenito, M. T. Garland, M. Fuentealba, A.H. Klahn, C. Manzur

**Affiliations:** a Laboratorio de Química Inorgánica, Instituto de Química, Facultad de Ciencias, Pontificia Universidad Católica de Valparaíso, Valparaíso, Chile; b Laboratorio de Cristalografía, Departamento de Física, Facultad de Ciencias Físicas y Matemáticas, Universidad de Chile, Santiago, Chile; c Universidad Andres Bello, Departamento de Ciencias Químicas, Santiago, Chile; d Laboratorio de Organometálica, Instituto de Química, Facultad de Ciencias, Pontificia Universidad Católica de Valparaíso, Valparaíso, Chile

## Abstract

In the title compound, [Fe(C_5_H_5_)(C_15_H_13_BrNO)], formed from the reaction of ferrocenoylacetone and 4-bromo­aniline, the mol­ecular structure is stabilized by an intra­molecular N—H⋯O hydrogen bond between the amine and carbonyl groups.

## Related literature

For related structures, see: Fuentealba *et al.* (2008 ▶); Shi *et al.* (2004 ▶, 2005 ▶, 2008 ▶). For the use of ferrocenes containing enamino­nes in the formation of transition metal complexes for olefin polymerization catalysts, see: Ye *et al.* (2008 ▶).
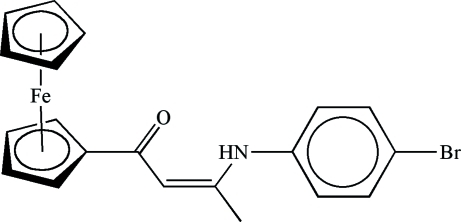


## Experimental

### 

#### Crystal data

[Fe(C_5_H_5_)(C_15_H_13_BrNO)]
*M* *_r_* = 424.11
Monoclinic, 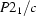

*a* = 16.808 (2) Å
*b* = 9.2589 (13) Å
*c* = 11.1133 (15) Å
β = 93.748 (2)°
*V* = 1725.8 (4) Å^3^
*Z* = 4
Mo *K*α radiation
μ = 3.20 mm^−1^
*T* = 298 K
0.30 × 0.20 × 0.03 mm

#### Data collection

Bruker SMART CCD area-detector diffractometer
Absorption correction: multi-scan (*SADABS*; Bruker, 2000 ▶) *T*_min_ = 0.684, *T*_max_ = 0.909
18029 measured reflections
3891 independent reflections
2709 reflections with *I* > 2σ(*I*)
*R*_int_ = 0.039

#### Refinement

*R*[*F* ^2^ > 2σ(*F* ^2^)] = 0.042
*wR*(*F* ^2^) = 0.102
*S* = 1.04
3891 reflections
222 parameters
H atoms treated by a mixture of independent and constrained refinement
Δρ_max_ = 0.69 e Å^−3^
Δρ_min_ = −0.24 e Å^−3^

### 

Data collection: *SMART-NT* (Bruker, 2001 ▶); cell refinement: *SAINT-NT* (Bruker, 2000 ▶); data reduction: *SAINT-NT*; program(s) used to solve structure: *SHELXS97* (Sheldrick, 2008 ▶); program(s) used to refine structure: *SHELXL97* (Sheldrick, 2008 ▶); molecular graphics: *OLEX2* (Dolomanov *et al.*, 2009 ▶); software used to prepare material for publication: *OLEX2*.

## Supplementary Material

Crystal structure: contains datablocks I, global. DOI: 10.1107/S1600536810047628/zq2074sup1.cif

Structure factors: contains datablocks I. DOI: 10.1107/S1600536810047628/zq2074Isup2.hkl

Additional supplementary materials:  crystallographic information; 3D view; checkCIF report

## Figures and Tables

**Table 1:** Hydrogen-bond geometry (Å, °)

*D*—H⋯*A*	*D*—H	H⋯*A*	*D*⋯*A*	*D*—H⋯*A*
N1—H1*N*⋯O1	0.74 (3)	1.98 (3)	2.612 (4)	143 (3)

**Fig. 1.**
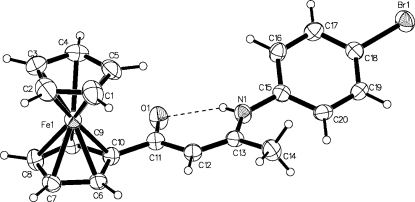
The molecular structure of the title compound with the atom numbering scheme. Displacement ellipsoids are presented at 30% probability level. H atoms are shown as a small spheres of arbitrary radius.

## supplementary crystallographic information

### Comment

Ferrocenes containing enaminones have attracted interest because they can be used as good chelating ligands for transition metals (Shi *et al.*, 2004, 2005, 2008). On the other hand, anions generated from these molecules offer alternatives to conventional ligands (cyclopentadienyl anions) in the formation of transition metal complexes for olefin polymerization catalysts (Ye *et al.*, 2008).

The title compound, [Fe(C_5_H_5_)(C_15_H_13_BrNO)], (I), is a Schiff base molecule resulting from the reaction of ferrocenoylacetone and 4-bromoaniline. It crystallizes in the monoclinic space group *P*2_1_/c and its asymmetric unit contains only one title molecule (Fig. 1).

The distances between the cyclopentadienyl ring centroids and the Fe atom are 1.644 Å and 1.634 Å, respectively. The cyclopentadienyl rings of the ferrocenyl group are parallel with a dihedral angle of 1.9 (3)° between the corresponding least-squares planes. These metrical parameters are typical of the η^5^···Fe···η^5^ coordination of the ferrocenyl moiety. The O1—C11—C12—C13—N1 skeleton is coplanar (rms deviation = 0.005 Å). The C≐O, C≐C and C≐N bond lengths are intermediate between their corresponding double and single bonds indicating that the π-system is partially delocalized in the framework. Likewise, the skeleton is nearly coplanar with the C_5_H_4_ ring with an angle of 6.9 (2)° between the mean planes. On the other hand, the angle between the least-squares planes of O1—C11—C12—C13—N1 and the bromophenyl ring C16/C20 is 33.8 (1)° out of the plane showing the lack of conjugation between both systems.

The molecular structure of the enaminone-containing compound is also stabilized by an intramolecular N-H···O hydrogen bond between the amine and the carbonyl groups (Table 1). This feature has been previously described by Fuentealba *et al.* (2008).

### Experimental

A solution of ferrocenoylacetone (0.2 g, 0.74 mmol), *p*-bromoaniline (0.13 g, 0.74 mmol) and a catalytic trace amount of *p*-TsOH in 30 ml of toluene was refluxed with a Dean-Stark apparatus to remove water for 12 h. The solvent was evaporated under vacuum, and the crude reaction mixture was submitted to column chromatography (silica gel 60, hexane:diethyl ether = 1:1 *v*/*v*). The title compound crystallizes from the solvent mixture to give red crystals (60%), mp. 168.9–171.0 °C.

IR (KBr) cm-1: 3079 (NH), 1606 (C=O), 1570 (C=C).

^1^H-NMR (399.95 MHz, CDCl_3_, 300 K): δ 12.66 (s, 1H, NH); 7.45 (d, 2H, J = 8 Hz; C_6_H_4_); 7.03 (d, 2H, J = 8 Hz; C_6_H_4_); 6.45 (s, 1H, CH); 4.78 (s, 2H, 2(H2, H5) of C_5_H_4_ ring); 4.44 (s, 2H, 2 (H3, H4) of C_5_H_4_ ring); 4.21 (s, 5H, C_5_H_5_); 2.12 (s, 3H, CH_3_).

^13^C-NMR (399.95 MHz, CDCl_3_, 300 K): δ 20.39; 68.47; 69.93; 70.17; 71.14; 96.99; 118.00; 125.55; 132.11; 138.22; 158.39; 192.47.

### Refinement

The N*H* hydrogen atom was located in a difference Fourier map and geometrically refined with *U*_iso_(H) = 1.2*U*_eq_(N). The other H atoms positions were calculated after each cycle of refinement using a riding model with C—H distances in the range 0.95—0.98 Å and *U*_iso_(H) = 1.2*U*_eq_(C).

### Crystal data

**Table:**

[Fe(C_5_H_5_)(C_15_H_13_BrNO)]	*F*(000) = 856
*M**_r_* = 424.11	*D*_x_ = 1.632 Mg m^−^^3^
Monoclinic, *P*2_1_/*c*	Mo *K*α radiation, λ = 0.71073 Å
Hall symbol: -P 2ybc	Cell parameters from 3891 reflections
*a* = 16.808 (2) Å	θ = 2.4–27.9°
*b* = 9.2589 (13) Å	µ = 3.20 mm^−^^1^
*c* = 11.1133 (15) Å	*T* = 298 K
β = 93.748 (2)°	Plate, red
*V* = 1725.8 (4) Å^3^	0.30 × 0.20 × 0.03 mm
*Z* = 4	

### Data collection

**Table:**

Bruker SMART CCD area-detector diffractometer	3891 independent reflections
Radiation source: fine-focus sealed tube	2709 reflections with *I* > 2σ(*I*)
graphite	*R*_int_ = 0.039
phi and ω scans	θ_max_ = 27.9°, θ_min_ = 2.4°
Absorption correction: multi-scan (SABABS; Bruker, 2000)	*h* = −21→20
*T*_min_ = 0.684, *T*_max_ = 0.909	*k* = −12→12
18029 measured reflections	*l* = −14→14

### Refinement

**Table:**

Refinement on *F*^2^	Primary atom site location: structure-invariant direct methods
Least-squares matrix: full	Secondary atom site location: difference Fourier map
*R*[*F*^2^ > 2σ(*F*^2^)] = 0.042	Hydrogen site location: inferred from neighbouring sites
*wR*(*F*^2^) = 0.102	H atoms treated by a mixture of independent and constrained refinement
*S* = 1.04	*w* = 1/[σ^2^(*F*_o_^2^) + (0.0515*P*)^2^ + 0.053*P*] where *P* = (*F*_o_^2^ + 2*F*_c_^2^)/3
3891 reflections	(Δ/σ)_max_ = 0.001
222 parameters	Δρ_max_ = 0.69 e Å^−^^3^
0 restraints	Δρ_min_ = −0.24 e Å^−^^3^

### Special details

**Table:**

Geometry. All e.s.d.'s (except the e.s.d. in the dihedral angle between two l.s. planes) are estimated using the full covariance matrix. The cell e.s.d.'s are taken into account individually in the estimation of e.s.d.'s in distances, angles and torsion angles; correlations between e.s.d.'s in cell parameters are only used when they are defined by crystal symmetry. An approximate (isotropic) treatment of cell e.s.d.'s is used for estimating e.s.d.'s involving l.s. planes.
Refinement. Refinement of *F*^2^ against ALL reflections. The weighted *R*-factor *wR* and goodness of fit *S* are based on *F*^2^, conventional *R*-factors *R* are based on *F*, with *F* set to zero for negative *F*^2^. The threshold expression of *F*^2^ > σ(*F*^2^) is used only for calculating *R*-factors(gt) *etc*. and is not relevant to the choice of reflections for refinement. *R*-factors based on *F*^2^ are statistically about twice as large as those based on *F*, and *R*- factors based on ALL data will be even larger.

### Fractional atomic coordinates and isotropic or equivalent isotropic displacement parameters (Å^2^)

**Table:**

	*x*	*y*	*z*	*U*_iso_*/*U*_eq_	
Fe1	0.87876 (2)	0.44531 (5)	0.71956 (4)	0.04981 (15)	
N1	0.70801 (17)	0.9570 (3)	0.6282 (3)	0.0521 (7)	
O1	0.82783 (14)	0.7961 (2)	0.57247 (18)	0.0644 (6)	
Br1	0.44949 (2)	1.32430 (4)	0.37244 (3)	0.06775 (15)	
C1	0.7855 (2)	0.3481 (4)	0.7956 (5)	0.0849 (12)	
H1	0.7677	0.3664	0.8765	0.102*	
C2	0.8421 (2)	0.2434 (4)	0.7644 (5)	0.0922 (14)	
H2	0.8710	0.1775	0.8208	0.111*	
C3	0.8512 (3)	0.2511 (5)	0.6407 (5)	0.0971 (14)	
H3	0.8867	0.1914	0.5950	0.117*	
C4	0.7998 (3)	0.3613 (5)	0.5930 (5)	0.0985 (15)	
H4	0.7933	0.3908	0.5083	0.118*	
C5	0.7592 (2)	0.4203 (4)	0.6888 (5)	0.0853 (12)	
H5	0.7197	0.4983	0.6822	0.102*	
C6	0.92063 (18)	0.6004 (3)	0.8360 (3)	0.0509 (7)	
H6	0.8977	0.6281	0.9115	0.061*	
C7	0.98002 (19)	0.4947 (4)	0.8225 (3)	0.0562 (8)	
H7	1.0044	0.4346	0.8875	0.067*	
C8	0.99673 (19)	0.4862 (4)	0.7004 (3)	0.0601 (8)	
H8	1.0349	0.4205	0.6659	0.072*	
C9	0.9482 (2)	0.5886 (3)	0.6363 (3)	0.0594 (8)	
H9	0.9470	0.6064	0.5493	0.071*	
C10	0.89992 (18)	0.6594 (3)	0.7192 (2)	0.0473 (7)	
C11	0.83605 (17)	0.7636 (3)	0.6819 (2)	0.0479 (7)	
C12	0.78603 (18)	0.8204 (3)	0.7693 (2)	0.0478 (7)	
H12	0.7966	0.7932	0.8493	0.057*	
C13	0.72388 (18)	0.9119 (3)	0.7432 (3)	0.0476 (7)	
C14	0.6714 (2)	0.9600 (4)	0.8393 (3)	0.0660 (9)	
H14A	0.6818	0.9018	0.9101	0.099*	
H14B	0.6165	0.9496	0.8109	0.099*	
H14C	0.6821	1.0594	0.8586	0.099*	
C15	0.64716 (17)	1.0437 (3)	0.5741 (3)	0.0462 (7)	
C16	0.61831 (19)	1.0096 (3)	0.4571 (3)	0.0526 (7)	
H16	0.6379	0.9287	0.4193	0.063*	
C17	0.5611 (2)	1.0941 (3)	0.3965 (3)	0.0547 (8)	
H17	0.5428	1.0709	0.3181	0.066*	
C18	0.53144 (17)	1.2125 (3)	0.4526 (3)	0.0489 (7)	
C19	0.55883 (19)	1.2483 (3)	0.5681 (3)	0.0582 (8)	
H19	0.5382	1.3285	0.6057	0.070*	
C20	0.6166 (2)	1.1656 (3)	0.6280 (3)	0.0591 (8)	
H20	0.6356	1.1915	0.7055	0.071*	
H1N	0.7336 (16)	0.923 (3)	0.585 (2)	0.027 (8)*	

### Atomic displacement parameters (Å^2^)

**Table:**

	*U*^11^	*U*^22^	*U*^33^	*U*^12^	*U*^13^	*U*^23^
Fe1	0.0471 (3)	0.0521 (3)	0.0496 (3)	0.00341 (19)	−0.00152 (19)	−0.00255 (19)
N1	0.0476 (16)	0.0602 (17)	0.0490 (15)	0.0069 (13)	0.0072 (13)	0.0016 (13)
O1	0.0722 (15)	0.0786 (15)	0.0436 (12)	0.0215 (12)	0.0128 (10)	0.0156 (10)
Br1	0.0698 (3)	0.0619 (2)	0.0704 (3)	0.00955 (16)	−0.00483 (18)	0.01312 (16)
C1	0.060 (2)	0.073 (3)	0.123 (4)	−0.004 (2)	0.016 (2)	0.011 (2)
C2	0.070 (3)	0.056 (2)	0.148 (4)	−0.008 (2)	−0.009 (3)	0.018 (3)
C3	0.078 (3)	0.073 (3)	0.137 (4)	0.002 (2)	−0.017 (3)	−0.043 (3)
C4	0.086 (3)	0.094 (3)	0.109 (4)	0.001 (3)	−0.037 (3)	−0.032 (3)
C5	0.048 (2)	0.069 (2)	0.136 (4)	0.0001 (18)	−0.019 (2)	−0.006 (3)
C6	0.0528 (18)	0.0598 (19)	0.0396 (15)	0.0030 (14)	−0.0014 (13)	−0.0050 (13)
C7	0.0540 (19)	0.0648 (19)	0.0488 (17)	0.0059 (16)	−0.0052 (14)	0.0010 (15)
C8	0.0494 (19)	0.067 (2)	0.065 (2)	0.0123 (16)	0.0116 (15)	0.0027 (16)
C9	0.062 (2)	0.069 (2)	0.0486 (18)	0.0074 (17)	0.0140 (15)	0.0071 (15)
C10	0.0490 (17)	0.0487 (17)	0.0448 (16)	0.0016 (13)	0.0069 (13)	0.0013 (12)
C11	0.0520 (17)	0.0495 (17)	0.0425 (16)	−0.0025 (14)	0.0056 (13)	0.0051 (13)
C12	0.0528 (18)	0.0530 (17)	0.0375 (15)	0.0023 (14)	0.0026 (13)	0.0025 (12)
C13	0.0447 (17)	0.0530 (17)	0.0449 (16)	−0.0050 (14)	0.0019 (12)	−0.0032 (13)
C14	0.062 (2)	0.086 (3)	0.0504 (18)	0.0137 (18)	0.0083 (16)	0.0009 (16)
C15	0.0434 (16)	0.0484 (17)	0.0470 (16)	−0.0050 (13)	0.0042 (13)	0.0041 (13)
C16	0.064 (2)	0.0504 (17)	0.0443 (16)	0.0076 (15)	0.0092 (14)	0.0006 (13)
C17	0.070 (2)	0.0575 (19)	0.0373 (15)	−0.0004 (16)	0.0050 (14)	0.0023 (13)
C18	0.0474 (17)	0.0437 (16)	0.0556 (18)	−0.0036 (13)	0.0024 (14)	0.0087 (13)
C19	0.065 (2)	0.0463 (17)	0.062 (2)	0.0037 (15)	−0.0035 (16)	−0.0114 (15)
C20	0.063 (2)	0.0502 (18)	0.0613 (19)	−0.0022 (16)	−0.0139 (16)	−0.0097 (15)

### Geometric parameters (Å, °)

**Table:**

Fe1—C10	2.014 (3)	C6—C10	1.430 (4)
Fe1—C4	2.024 (4)	C6—H6	0.9800
Fe1—C6	2.028 (3)	C7—C8	1.405 (4)
Fe1—C9	2.029 (3)	C7—H7	0.9800
Fe1—C5	2.030 (4)	C8—C9	1.413 (4)
Fe1—C1	2.038 (4)	C8—H8	0.9800
Fe1—C7	2.040 (3)	C9—C10	1.426 (4)
Fe1—C3	2.040 (4)	C9—H9	0.9800
Fe1—C2	2.041 (4)	C10—C11	1.482 (4)
Fe1—C8	2.043 (3)	C11—C12	1.426 (4)
N1—C13	1.355 (4)	C12—C13	1.361 (4)
N1—C15	1.404 (4)	C12—H12	0.9300
N1—H1N	0.74 (3)	C13—C14	1.497 (4)
O1—C11	1.252 (3)	C14—H14A	0.9600
Br1—C18	1.898 (3)	C14—H14B	0.9600
C1—C5	1.408 (6)	C14—H14C	0.9600
C1—C2	1.418 (6)	C15—C20	1.392 (4)
C1—H1	0.9800	C15—C16	1.394 (4)
C2—C3	1.395 (6)	C16—C17	1.380 (4)
C2—H2	0.9800	C16—H16	0.9300
C3—C4	1.416 (6)	C17—C18	1.371 (4)
C3—H3	0.9800	C17—H17	0.9300
C4—C5	1.412 (6)	C18—C19	1.375 (4)
C4—H4	0.9800	C19—C20	1.374 (4)
C5—H5	0.9800	C19—H19	0.9300
C6—C7	1.413 (4)	C20—H20	0.9300
			
C10—Fe1—C4	119.00 (16)	Fe1—C4—H4	126.0
C10—Fe1—C6	41.43 (12)	C1—C5—C4	108.1 (4)
C4—Fe1—C6	154.10 (16)	C1—C5—Fe1	70.1 (2)
C10—Fe1—C9	41.31 (12)	C4—C5—Fe1	69.4 (2)
C4—Fe1—C9	107.74 (19)	C1—C5—H5	125.9
C6—Fe1—C9	69.03 (12)	C4—C5—H5	125.9
C10—Fe1—C5	106.56 (14)	Fe1—C5—H5	125.9
C4—Fe1—C5	40.76 (18)	C7—C6—C10	107.4 (3)
C6—Fe1—C5	119.11 (16)	C7—C6—Fe1	70.11 (18)
C9—Fe1—C5	126.17 (16)	C10—C6—Fe1	68.73 (16)
C10—Fe1—C1	125.17 (14)	C7—C6—H6	126.3
C4—Fe1—C1	68.4 (2)	C10—C6—H6	126.3
C6—Fe1—C1	106.98 (16)	Fe1—C6—H6	126.3
C9—Fe1—C1	163.40 (15)	C8—C7—C6	109.2 (3)
C5—Fe1—C1	40.51 (17)	C8—C7—Fe1	70.00 (18)
C10—Fe1—C7	68.85 (13)	C6—C7—Fe1	69.25 (17)
C4—Fe1—C7	163.89 (17)	C8—C7—H7	125.4
C6—Fe1—C7	40.64 (12)	C6—C7—H7	125.4
C9—Fe1—C7	68.01 (13)	Fe1—C7—H7	125.4
C5—Fe1—C7	154.25 (17)	C7—C8—C9	107.7 (3)
C1—Fe1—C7	120.16 (17)	C7—C8—Fe1	69.73 (18)
C10—Fe1—C3	154.39 (19)	C9—C8—Fe1	69.17 (19)
C4—Fe1—C3	40.77 (17)	C7—C8—H8	126.2
C6—Fe1—C3	163.30 (18)	C9—C8—H8	126.2
C9—Fe1—C3	120.10 (19)	Fe1—C8—H8	126.2
C5—Fe1—C3	68.43 (17)	C8—C9—C10	108.4 (3)
C1—Fe1—C3	68.3 (2)	C8—C9—Fe1	70.23 (19)
C7—Fe1—C3	126.63 (16)	C10—C9—Fe1	68.76 (17)
C10—Fe1—C2	163.44 (18)	C8—C9—H9	125.8
C4—Fe1—C2	67.9 (2)	C10—C9—H9	125.8
C6—Fe1—C2	126.21 (18)	Fe1—C9—H9	125.8
C9—Fe1—C2	154.43 (17)	C9—C10—C6	107.2 (3)
C5—Fe1—C2	68.00 (16)	C9—C10—C11	123.4 (3)
C1—Fe1—C2	40.68 (16)	C6—C10—C11	129.1 (3)
C7—Fe1—C2	108.82 (16)	C9—C10—Fe1	69.93 (17)
C3—Fe1—C2	39.98 (18)	C6—C10—Fe1	69.84 (17)
C10—Fe1—C8	69.19 (13)	C11—C10—Fe1	121.1 (2)
C4—Fe1—C8	126.54 (18)	O1—C11—C12	122.7 (3)
C6—Fe1—C8	68.69 (13)	O1—C11—C10	117.4 (3)
C9—Fe1—C8	40.61 (12)	C12—C11—C10	119.9 (2)
C5—Fe1—C8	163.86 (18)	C13—C12—C11	124.3 (3)
C1—Fe1—C8	154.51 (16)	C13—C12—H12	117.9
C7—Fe1—C8	40.27 (12)	C11—C12—H12	117.9
C3—Fe1—C8	108.27 (17)	N1—C13—C12	119.6 (3)
C2—Fe1—C8	120.36 (15)	N1—C13—C14	119.5 (3)
C13—N1—C15	132.3 (3)	C12—C13—C14	120.9 (3)
C13—N1—H1N	113 (2)	C13—C14—H14A	109.5
C15—N1—H1N	114 (2)	C13—C14—H14B	109.5
C5—C1—C2	107.3 (4)	H14A—C14—H14B	109.5
C5—C1—Fe1	69.4 (2)	C13—C14—H14C	109.5
C2—C1—Fe1	69.7 (2)	H14A—C14—H14C	109.5
C5—C1—H1	126.3	H14B—C14—H14C	109.5
C2—C1—H1	126.3	C20—C15—C16	118.0 (3)
Fe1—C1—H1	126.3	C20—C15—N1	123.9 (3)
C3—C2—C1	108.9 (4)	C16—C15—N1	118.0 (3)
C3—C2—Fe1	70.0 (2)	C17—C16—C15	121.0 (3)
C1—C2—Fe1	69.6 (2)	C17—C16—H16	119.5
C3—C2—H2	125.5	C15—C16—H16	119.5
C1—C2—H2	125.5	C18—C17—C16	119.6 (3)
Fe1—C2—H2	125.5	C18—C17—H17	120.2
C2—C3—C4	107.6 (4)	C16—C17—H17	120.2
C2—C3—Fe1	70.0 (2)	C17—C18—C19	120.5 (3)
C4—C3—Fe1	69.0 (2)	C17—C18—Br1	119.8 (2)
C2—C3—H3	126.2	C19—C18—Br1	119.7 (2)
C4—C3—H3	126.2	C20—C19—C18	120.0 (3)
Fe1—C3—H3	126.2	C20—C19—H19	120.0
C5—C4—C3	108.1 (4)	C18—C19—H19	120.0
C5—C4—Fe1	69.8 (2)	C19—C20—C15	120.8 (3)
C3—C4—Fe1	70.2 (2)	C19—C20—H20	119.6
C5—C4—H4	126.0	C15—C20—H20	119.6
C3—C4—H4	126.0		
			
C10—Fe1—C1—C5	73.3 (3)	C10—Fe1—C7—C8	−82.4 (2)
C4—Fe1—C1—C5	−37.8 (3)	C4—Fe1—C7—C8	39.9 (7)
C6—Fe1—C1—C5	115.2 (3)	C6—Fe1—C7—C8	−120.8 (3)
C9—Fe1—C1—C5	41.8 (7)	C9—Fe1—C7—C8	−37.81 (19)
C7—Fe1—C1—C5	157.5 (2)	C5—Fe1—C7—C8	−166.7 (3)
C3—Fe1—C1—C5	−81.8 (3)	C1—Fe1—C7—C8	158.3 (2)
C2—Fe1—C1—C5	−118.5 (4)	C3—Fe1—C7—C8	74.1 (3)
C8—Fe1—C1—C5	−168.8 (3)	C2—Fe1—C7—C8	115.1 (2)
C10—Fe1—C1—C2	−168.3 (3)	C10—Fe1—C7—C6	38.41 (18)
C4—Fe1—C1—C2	80.7 (3)	C4—Fe1—C7—C6	160.7 (6)
C6—Fe1—C1—C2	−126.3 (3)	C9—Fe1—C7—C6	83.0 (2)
C9—Fe1—C1—C2	160.3 (5)	C5—Fe1—C7—C6	−45.9 (4)
C5—Fe1—C1—C2	118.5 (4)	C1—Fe1—C7—C6	−80.9 (2)
C7—Fe1—C1—C2	−84.1 (3)	C3—Fe1—C7—C6	−165.1 (3)
C3—Fe1—C1—C2	36.7 (3)	C2—Fe1—C7—C6	−124.1 (2)
C8—Fe1—C1—C2	−50.3 (5)	C8—Fe1—C7—C6	120.8 (3)
C5—C1—C2—C3	0.4 (5)	C6—C7—C8—C9	0.7 (4)
Fe1—C1—C2—C3	−59.1 (3)	Fe1—C7—C8—C9	59.0 (2)
C5—C1—C2—Fe1	59.5 (3)	C6—C7—C8—Fe1	−58.3 (2)
C10—Fe1—C2—C3	156.0 (5)	C10—Fe1—C8—C7	81.5 (2)
C4—Fe1—C2—C3	38.1 (3)	C4—Fe1—C8—C7	−167.2 (2)
C6—Fe1—C2—C3	−167.0 (2)	C6—Fe1—C8—C7	36.91 (19)
C9—Fe1—C2—C3	−46.9 (5)	C9—Fe1—C8—C7	119.1 (3)
C5—Fe1—C2—C3	82.2 (3)	C5—Fe1—C8—C7	159.0 (5)
C1—Fe1—C2—C3	120.2 (4)	C1—Fe1—C8—C7	−47.9 (4)
C7—Fe1—C2—C3	−125.1 (3)	C3—Fe1—C8—C7	−125.6 (3)
C8—Fe1—C2—C3	−82.4 (3)	C2—Fe1—C8—C7	−83.5 (3)
C10—Fe1—C2—C1	35.7 (7)	C10—Fe1—C8—C9	−37.69 (18)
C4—Fe1—C2—C1	−82.2 (3)	C4—Fe1—C8—C9	73.6 (3)
C6—Fe1—C2—C1	72.8 (3)	C6—Fe1—C8—C9	−82.2 (2)
C9—Fe1—C2—C1	−167.1 (3)	C5—Fe1—C8—C9	39.8 (6)
C5—Fe1—C2—C1	−38.0 (3)	C1—Fe1—C8—C9	−167.1 (3)
C7—Fe1—C2—C1	114.7 (3)	C7—Fe1—C8—C9	−119.1 (3)
C3—Fe1—C2—C1	−120.2 (4)	C3—Fe1—C8—C9	115.2 (2)
C8—Fe1—C2—C1	157.4 (3)	C2—Fe1—C8—C9	157.4 (2)
C1—C2—C3—C4	−0.1 (5)	C7—C8—C9—C10	−1.0 (4)
Fe1—C2—C3—C4	−59.0 (3)	Fe1—C8—C9—C10	58.3 (2)
C1—C2—C3—Fe1	58.8 (3)	C7—C8—C9—Fe1	−59.3 (2)
C10—Fe1—C3—C2	−164.4 (3)	C10—Fe1—C9—C8	120.0 (3)
C4—Fe1—C3—C2	−119.0 (4)	C4—Fe1—C9—C8	−126.0 (2)
C6—Fe1—C3—C2	39.1 (7)	C6—Fe1—C9—C8	81.3 (2)
C9—Fe1—C3—C2	158.6 (2)	C5—Fe1—C9—C8	−167.3 (2)
C5—Fe1—C3—C2	−81.1 (3)	C1—Fe1—C9—C8	160.3 (5)
C1—Fe1—C3—C2	−37.3 (3)	C7—Fe1—C9—C8	37.50 (19)
C7—Fe1—C3—C2	74.9 (3)	C3—Fe1—C9—C8	−83.1 (2)
C8—Fe1—C3—C2	115.8 (3)	C2—Fe1—C9—C8	−50.3 (4)
C10—Fe1—C3—C4	−45.4 (5)	C4—Fe1—C9—C10	114.0 (2)
C6—Fe1—C3—C4	158.1 (5)	C6—Fe1—C9—C10	−38.71 (18)
C9—Fe1—C3—C4	−82.4 (3)	C5—Fe1—C9—C10	72.7 (3)
C5—Fe1—C3—C4	37.9 (3)	C1—Fe1—C9—C10	40.3 (6)
C1—Fe1—C3—C4	81.7 (3)	C7—Fe1—C9—C10	−82.53 (19)
C7—Fe1—C3—C4	−166.2 (3)	C3—Fe1—C9—C10	156.8 (2)
C2—Fe1—C3—C4	119.0 (4)	C2—Fe1—C9—C10	−170.4 (4)
C8—Fe1—C3—C4	−125.3 (3)	C8—Fe1—C9—C10	−120.0 (3)
C2—C3—C4—C5	−0.2 (5)	C8—C9—C10—C6	1.0 (4)
Fe1—C3—C4—C5	−59.8 (3)	Fe1—C9—C10—C6	60.2 (2)
C2—C3—C4—Fe1	59.6 (3)	C8—C9—C10—C11	−173.9 (3)
C10—Fe1—C4—C5	−81.7 (3)	Fe1—C9—C10—C11	−114.7 (3)
C6—Fe1—C4—C5	−46.9 (6)	C8—C9—C10—Fe1	−59.2 (2)
C9—Fe1—C4—C5	−125.3 (3)	C7—C6—C10—C9	−0.6 (4)
C1—Fe1—C4—C5	37.5 (3)	Fe1—C6—C10—C9	−60.2 (2)
C7—Fe1—C4—C5	162.7 (5)	C7—C6—C10—C11	173.9 (3)
C3—Fe1—C4—C5	118.9 (4)	Fe1—C6—C10—C11	114.2 (3)
C2—Fe1—C4—C5	81.5 (3)	C7—C6—C10—Fe1	59.7 (2)
C8—Fe1—C4—C5	−166.3 (2)	C4—Fe1—C10—C9	−84.1 (3)
C10—Fe1—C4—C3	159.4 (3)	C6—Fe1—C10—C9	118.0 (3)
C6—Fe1—C4—C3	−165.8 (4)	C5—Fe1—C10—C9	−126.5 (2)
C9—Fe1—C4—C3	115.8 (3)	C1—Fe1—C10—C9	−166.9 (2)
C5—Fe1—C4—C3	−118.9 (4)	C7—Fe1—C10—C9	80.3 (2)
C1—Fe1—C4—C3	−81.4 (3)	C3—Fe1—C10—C9	−52.0 (4)
C7—Fe1—C4—C3	43.8 (8)	C2—Fe1—C10—C9	165.3 (5)
C2—Fe1—C4—C3	−37.4 (3)	C8—Fe1—C10—C9	37.07 (18)
C8—Fe1—C4—C3	74.8 (4)	C4—Fe1—C10—C6	157.9 (2)
C2—C1—C5—C4	−0.5 (4)	C9—Fe1—C10—C6	−118.0 (3)
Fe1—C1—C5—C4	59.2 (3)	C5—Fe1—C10—C6	115.5 (2)
C2—C1—C5—Fe1	−59.7 (3)	C1—Fe1—C10—C6	75.0 (2)
C3—C4—C5—C1	0.5 (5)	C7—Fe1—C10—C6	−37.71 (18)
Fe1—C4—C5—C1	−59.6 (3)	C3—Fe1—C10—C6	−170.0 (3)
C3—C4—C5—Fe1	60.1 (3)	C2—Fe1—C10—C6	47.3 (6)
C10—Fe1—C5—C1	−125.2 (2)	C8—Fe1—C10—C6	−80.97 (19)
C4—Fe1—C5—C1	119.3 (4)	C4—Fe1—C10—C11	33.6 (3)
C6—Fe1—C5—C1	−82.1 (3)	C6—Fe1—C10—C11	−124.3 (3)
C9—Fe1—C5—C1	−166.4 (2)	C9—Fe1—C10—C11	117.7 (3)
C7—Fe1—C5—C1	−49.7 (4)	C5—Fe1—C10—C11	−8.8 (3)
C3—Fe1—C5—C1	81.4 (3)	C1—Fe1—C10—C11	−49.3 (3)
C2—Fe1—C5—C1	38.2 (3)	C7—Fe1—C10—C11	−162.0 (3)
C8—Fe1—C5—C1	162.6 (5)	C3—Fe1—C10—C11	65.7 (4)
C10—Fe1—C5—C4	115.4 (3)	C2—Fe1—C10—C11	−77.0 (6)
C6—Fe1—C5—C4	158.6 (3)	C8—Fe1—C10—C11	154.7 (3)
C9—Fe1—C5—C4	74.3 (3)	C9—C10—C11—O1	−4.2 (5)
C1—Fe1—C5—C4	−119.3 (4)	C6—C10—C11—O1	−177.8 (3)
C7—Fe1—C5—C4	−169.0 (3)	Fe1—C10—C11—O1	−89.5 (3)
C3—Fe1—C5—C4	−37.9 (3)	C9—C10—C11—C12	174.7 (3)
C2—Fe1—C5—C4	−81.1 (3)	C6—C10—C11—C12	1.1 (5)
C8—Fe1—C5—C4	43.3 (6)	Fe1—C10—C11—C12	89.4 (3)
C10—Fe1—C6—C7	−118.9 (3)	O1—C11—C12—C13	1.6 (5)
C4—Fe1—C6—C7	−167.8 (4)	C10—C11—C12—C13	−177.2 (3)
C9—Fe1—C6—C7	−80.3 (2)	C15—N1—C13—C12	176.6 (3)
C5—Fe1—C6—C7	159.1 (2)	C15—N1—C13—C14	−1.7 (5)
C1—Fe1—C6—C7	116.8 (2)	C11—C12—C13—N1	−1.8 (5)
C3—Fe1—C6—C7	46.0 (7)	C11—C12—C13—C14	176.5 (3)
C2—Fe1—C6—C7	76.2 (2)	C13—N1—C15—C20	39.0 (5)
C8—Fe1—C6—C7	−36.58 (19)	C13—N1—C15—C16	−144.2 (3)
C4—Fe1—C6—C10	−49.0 (5)	C20—C15—C16—C17	−0.1 (5)
C9—Fe1—C6—C10	38.60 (18)	N1—C15—C16—C17	−177.1 (3)
C5—Fe1—C6—C10	−82.1 (2)	C15—C16—C17—C18	−0.8 (5)
C1—Fe1—C6—C10	−124.3 (2)	C16—C17—C18—C19	0.7 (5)
C7—Fe1—C6—C10	118.9 (3)	C16—C17—C18—Br1	−177.3 (2)
C3—Fe1—C6—C10	164.9 (6)	C17—C18—C19—C20	0.3 (5)
C2—Fe1—C6—C10	−165.0 (2)	Br1—C18—C19—C20	178.3 (2)
C8—Fe1—C6—C10	82.28 (19)	C18—C19—C20—C15	−1.2 (5)
C10—C6—C7—C8	−0.1 (4)	C16—C15—C20—C19	1.0 (5)
Fe1—C6—C7—C8	58.7 (2)	N1—C15—C20—C19	177.8 (3)
C10—C6—C7—Fe1	−58.8 (2)		

### Hydrogen-bond geometry (Å, °)

**Table:**

*D*—H···*A*	*D*—H	H···*A*	*D*···*A*	*D*—H···*A*
N1—H1N···O1	0.74 (3)	1.98 (3)	2.612 (4)	143 (3)
